# Hsp90 Inhibitors Prevent HSV-1 Replication by Directly Targeting UL42-Hsp90 Complex

**DOI:** 10.3389/fmicb.2021.797279

**Published:** 2022-02-03

**Authors:** Shurong Qin, Xiao Hu, Shimin Lin, Ji Xiao, Zhaoyang Wang, Jiaoyan Jia, Xiaowei Song, Kaisheng Liu, Zhe Ren, Yifei Wang

**Affiliations:** ^1^Guangzhou Jinan Biomedical Research and Development Center, College of Life Science and Technology, Institute of Biomedicine, Jinan University, Guangzhou, China; ^2^Key Laboratory of Virology of Guangzhou, Jinan University, Guangzhou, China; ^3^Key Laboratory of Bioengineering Medicine of Guangdong Province, Jinan University, Guangzhou, China; ^4^College of Pharmacy, Jinan University, Guangzhou, China; ^5^Shenzhen People’s Hospital (The Second Clinical Medical College, Jinan University, The First Affiliated Hospital, Southern University of Science and Technology), Shenzhen, China

**Keywords:** HSV-1, AT-533, 17-AAG, DNA replication, UL42, protein docking

## Abstract

Herpes simplex virus type I (HSV-1) is a member of the Alphaherpesvirinae family, which could initiate labial herpes caused by the reactivation of HSV-1 primary infection, and secondary infection even causes herpes encephalitis. The study presented here demonstrates that Hsp90 inhibitors (AT-533 and 17-AAG) directly targeted the HSV-1 UL42-Hsp90 complex, and Hsp90 interacted with HSV-1 UL42 in silicon and experiment. Interestingly, Hsp90 inhibitors also reduced virus titers of ACV-resistant clinical HSV-1 strains (153 and blue strain), revealing that HSV-1 UL42 would be a new target against ACV-resistant HSV-1 strains. Altogether, this present study indicates that Hsp90 inhibitors prevent HSV-1 proliferation by regulating the interaction between Hsp90 and HSV-1 UL42, suggesting a promising target for anti-HSV-1 therapies in the replication.

## Introduction

Herpes simplex virus-1 (HSV-1), a common human pathogenic virus, belongs to the α Herpesviridae family (Yoshikawa, 2000). HSV-1 could initiate labial herpes caused by the reactivation of HSV-1 primary infection, and secondary infection even causes herpes encephalitis (Kennedy et al., 2015). Specifically, HSV-1 contains at least 7 conserved proteins to regulate DNA replication, such as origin-binding protein UL9, single-stranded DNA binding protein ICP8, DNA polymerase complex UL30/42 and helicase-primase complex UL5/8/52 (James et al., 2015). Among the most important HSV-1 proteins, HSV-1 DNA polymerase and the early enzyme thymidine kinase (TK) play a key role in the virus replication process, so drugs targeting these proteins may disrupt the viral replication cycle (Souza et al., 2008). At present, nucleic acid analogs have been used as anti-HSV-1 drugs in the clinical frontline, which targets the stage of viral DNA replication, especially acting on TK. The representative drug is acyclovir and its derivatives. Due to the limitations of nucleoside analog drugs, long-term use of these drugs to treat HSV-1 infection may induce drug-resistant virus strains and adverse reactions (Piret and Boivin, 2011). Therefore, it is crucial to develop novel antiviral drugs targeting different life stages.

Recently, many studies have indicated that heat shock protein 90 (Hsp90) is a promising broad-spectrum antiviral host factor required by many viruses for multiple phases of their life cycle, including viral entry, nuclear import, transcription, and replication (Wang et al., 2017). Our previous studies clarified and confirmed the potent bioactivity of novel Hsp90 inhibitors, including AT-533, AT-760, and SNX-2112. AT-533 and AT-670 alleviated HSV-1 keratitis in a rabbit model (Xiang et al., 2012). Strikingly, AT-533 efficiently inhibited the nuclear egress of the viral nucleocapsid and the assembly of virus particles (Li et al., 2018). In addition, AT-533 attenuated HSV-1-induced inflammation through inhibiting the cleavage of pro-IL-1β to mature IL-1β (Li et al., 2019).

Previous research indicated that Hsp90 inhibitor geldanamycin reduced viral titers and induced the abnormal location of HSV-1 DNA polymerase catalytic subunit pUL30 (Burch and Weller, 2005). Moreover, Hsp90 interacted with the Epstein-Barr virus (EBV) DNA polymerase processivity factor BMRF1 in the cytoplasm to assist complex formation with polymerase catalytic subunit BALF5, indicating the interaction between Hsp90 and its client protein BMRF1 is vital for EBV genome synthesis and disease development (Kawashima et al., 2013). However, the particular interaction between Hsp90 and HSV-1 other replication-related proteins is still unknown. This study further studied the underlying mechanism of antiviral activity of Hsp90 inhibitor AT-533 and 17-AAG against HSV-1 DNA replication-related proteins and ACV-resistant strains.

## Materials and Methods

### Inhibitors, Antibodies, and Plasmids

AT-533 was systematically synthesized in a previously reported study (Li et al., 2018), and 17-N-allylamino 17-demethoxygeldanamycin (17-AAG) (S1141) was purchased from Selleck. Cell Counting Kit-8 (CCK-8) was obtained from Beyotime Biotechnology. Trizol Reagent was purchased from Invitrogen (Carlsbad, CA). Dulbecco’s modified Eagle medium (DMEM), fetal bovine serum (FBS), and penicillin-streptomycin were bought from Gibco-BRL (Gland Island, NY). Ubiquitin—proteasome inhibitor MG-132 was purchased from Selleck (S2619), and autophagy inhibitor chloroquine (CQ) was purchased from Sigma Aldrich (C6628). These compounds were dissolved into designated concentrations in dimethyl sulfoxide (DMSO), whose final concentrations were less than 0.1%. The primary antibodies used in this study included mouse monoclonal antibodies (MAb) against HSV-1 ICP8 (Santa Cruz,11E2), HSV-1 UL42 (Abcam, ab19311), LC3B (Cell Signaling Technology, 2775), and rabbit monoclonal antibodies (Mab) against Total Hsp90 (Abcam,ab13492), Ubiquitin-P4D1 (Cell Signaling Technology, 3936), Akt (Cell Signaling Technology, 13038S), GAPDH (GeneTek, GTX100118), Flag-tag (Cell Signaling Technology, 14793S), HA-tag (Cell Signaling Technology, 3724), and GFP-tag (Beyotime Biotechnology, AF0159). The secondary antibodies mainly included anti-rabbit IgG light chain (Abbkine, A25022) and anti-mouse IgG light chain (Abbkine, A25012). The eukaryotic expression plasmids, including pLVX-GFP-C1-UL30, pCMV-HA-UL42, were generated in our laboratory. RNA template was obtained from HSV-1-infected cells to amplify the virus coding sequence and then reverse transcripted to cDNA. All constructed plasmids were verified by DNA sequencing (TSINGKE Biological Technology). The primers and vectors used to construct the plasmids were listed in Supplementary Table 1.

### Cells and Viruses

Human foreskin fibroblast (ATCC SCRC1041), human embryonic kidney cells (HEK293T, ATCC CRL1573), and African green monkey kidney cells (Vero, ATCC CCL181) were cultured in Dulbecco’s modified Eagle’s medium (DMEM, Gibco) complemented with 10% fetal bovine serum (FBS, Gibco). The maintenance medium used for the dilutions of virus and reagents was DMEM complemented with 2% FBS. HSV-1/F (ATCC VR-733) was obtained from Hong Kong University. ACV-resistant clinical HSV-1 strain (HSV-1/153), a TK-mutant derived from HSV-1 (KOS)-HSV-1/Blue (Wang et al., 2011) were a kind present from Tao Peng (Guangzhou Institutes of Biomedicine and Health, Chinese Academy of Sciences). These above viruses were propagated in Vero cells and preserved at –80°C until use.

### CCK-8 Assay

The CCK-8 assay was manipulated according to the supplier’s protocol (Beyotime Biotechnology, C0037). Briefly, HFF cells were cultured in 96-wells plates and reached 85% cell confluence. Various concentrations of the compound were added to the plate, with each concentration having three replicates. After 48 h of incubation, 10 μL CCK-8 solution (5 mg/mL) was added to each well, and the plate was incubated for 1 h, protected from light. Then, the plates were incubated for 10 min at room temperature (RT) with gentle shaking. The optical density (OD) at 450 nm was measured with an enzyme-labeled reader (Bio-Rad, Hercules, CA). The 50% cytotoxicity concentration (CC_50_) was defined as reducing 50% cell viability.

### Viral Titer Determination

Vero cells were cultured in 96-wells plates. The next day, Vero cell monolayers were incubated with 11-fold serial dilutions of 100 μL HSV-1 treatment and incubated at 37°C and 5% CO_2_ for 72 h. Morphological changes of the cells (cytotoxic effects, CPEs) were observed every day under an inverted microscope, and cell viability was calculated based on CPEs. The control cells were treated only with maintenance medium DMEM. The plaque assay was carried out in triplicate, and the virus was quantified by serial dilution and titration assay. The TCID50 (50% tissue culture infectious dose) was calculated using the Reed and Muench method. Log10 50% endpoint dilutions = log10 of dilution showing a mortality next above 50% – (difference of logarithms × logarithm of dilution factor). Difference of logarithms = [(mortality at dilution next above 50%) – 50%]/[(mortality next above 50%) –(mortality next below 50%)] (Ramakrishnan, 2016).

### Antiviral Effect of GFP-HSV-1

HFF cells were cultured in 96-wells plates, and on the second day, cell monolayers were incubated with 11-fold serial dilutions of 100 μL Hsp90 inhibitor treatment and infected MOI = 1 GFP-HSV-1 at 37°C and 5% CO_2_ for 72 h (Wang et al., 2018). The plaque assay was carried out in triplicate. GFP fluorescence was quantified using a Synergy Neo HTS Multi-Mode Reader (Biotek Instruments). The relative fluorescence intensity was calculated using the following formula: Relative fluorescence intensity = (Hsp90 inhibitors treatment fluorescence intensity)/(GFP-HSV-1 control fluorescence intensity) × 100%.

### CPEs Cell Viability Assay

HFF cells were cultured in 96-well plates. The next day, HFF cell monolayers were incubated with 11-fold serial dilutions of 100 μL HSV-1 treated with or without Hsp90 inhibitors at 37°C and 5% CO_2_. After 72 h, 10 μL CCK-8 solution was added to each well and incubated for 1 h in the dark. Then, the plates were incubated at RT for 10 min with gentle shaking. The optical density (OD) at 450 nm was measured with an enzyme-labeled reader (Bio-Rad, Hercules, CA). The CPEs inhibition ratio was calculated by the formula as follows. The CPEs inhibition ratio = [(Absorption of treatment groups)–(Absorption of cell control)]/[(Absorption of virus control)–(Absorption of cell control)] × 100% (Bonvicini et al., 2018).

### Protein Docking Stimulation

ZDock^1^ was manipulated to predict the structures of protein-protein interaction and symmetric multimers. Prediction of protein-protein interaction was in three steps as follows (Pierce et al., 2014). First, input ligand and receptor protein structures and choose the ZDock version options; second, select the blocking/contacting residues; view docking results online or download in Pymol. The docking results were obtained from the binding affinity (ΔG) and dissociation constant (Kd) to predict protein-protein interactions value.

### Molecular Docking Simulation

LeDock^2^ was manipulated to simulate the docking between small molecular compounds and proteins. Docking binding affinity (ΔG) < 0 kJ/mol indicated ligand molecules could spontaneously bind to receptor proteins, while (ΔG) <5 kJ/mol indicated both of them can bind stably (He et al., 2019). The target proteins in all networks were obtained 3D structures from the RCSB PDB database^3^ (Burley et al., 2019). 2D structures of all compounds were obtained from ZINC^4^ (Sterling and Irwin, 2015). Top 10 docking complexes were docked between each protein and ligand and chosen the complexes with the smallest binding energy.

### Co-immunoprecipitation

HFF cells were infected with HSV-1 for 12 h in a 100 mm^2^ flask dish, which was collected and lysed in 100 μL SDS Lysis Buffer (Beyotime, China) containing 1% PMSF and centrifuged at 12,000 g. The supernatant was divided into two parts, one for input and the other was incubated with IgG (normal mouse or rabbit IgG, primary antibody) at 4°C overnight. Then the mixture was treated with 35 μL of the volume of protein A/G magnetic beads at 4°C for 2 h. Next, the immune-precipitates were collected, washed three times with PBS, and re-suspended in 30 μL 1 × SDS-PAGE buffer (Beyotime, China). Finally, the samples were boiled for 10 min and analyzed by Western blot.

### Plasmid Transfection

HEK293T cells were seeded in 12-well plates, and on the second day, the mentioned plasmids were performed with Lipofectamine-3000 transfection reagent according to the manufacturer’s instructions (Invitrogen). Briefly, 500 ng of the corresponding plasmids and Lipofectamine 3,000 reagent were diluted in 100 μL Opti-MEM reduced serum medium (Invitrogen). The diluted plasmids were then added to the Lipofectamine 3,000 (1:1 ratio), mixed, and incubated at RT for 10 min. The transfection mixture was then added to cells at 60–70% and was replaced with flesh 10% FBS DMEM after transfection for 6 h.

### Western Blot

HFF cells were seeded in 12-well plates with a density of 1.5 × 10^6^ cells/well. Until 85% cell confluence, cells were infected with HSV-1 (MOI = 20) at 37°C for 4 h. Therefore, DMEM maintenance medium with or without AT-533 or 17-AAG was added. At 12 h post-infection, the cells were washed three times with PBS and were lysed with 1*SDS-PAGE buffer (Beyotime). The equal amount (40 μg/sample) of proteins were subjected to Western blot analysis.

### Statistical Analysis

Research data were calculated as the mean ± SD, and unpaired Student’s *t*-test determined statistical significance. The statistical significance was determined by *P*-values (*P* < 0.05). *P*-values of <0.05, <0.01, and <0.005 were marked as *, **, and *** separately. Experiments were repeated 3 times, and the results were analyzed by GraphPad 7.0.

## Results

### Cytotoxicity and Anti-HSV-1 Activity of AT-533 and 17-AAG

**FIGURE 1:**
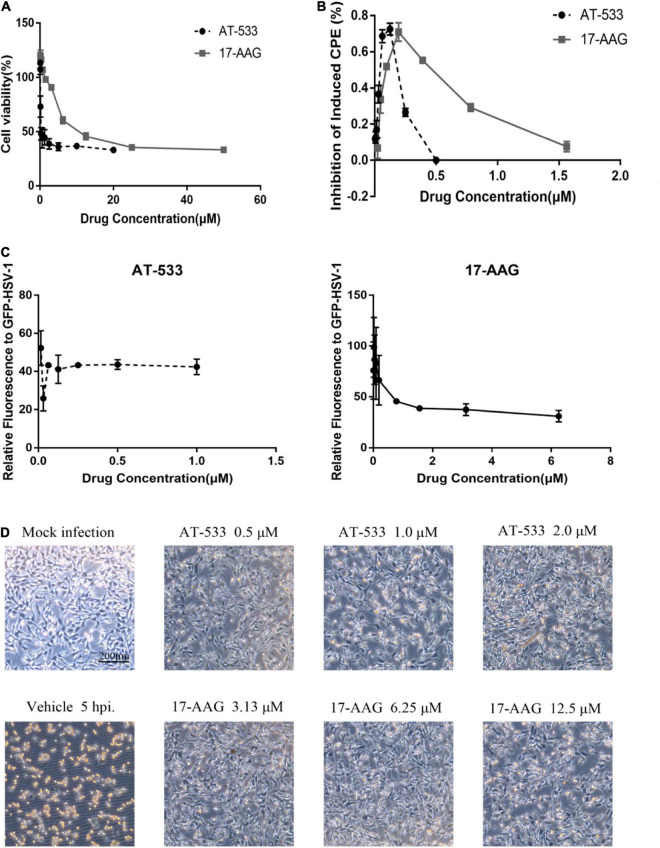
Cytotoxicity and anti-HSV-1 activity of AT-533 and 17-AAG. **(A)** Human foreskin fibroblast cell monolayers were treated with concentration-gradient AT-533 and 17-AAG for 48 h and were manipulated to CCK-8 assay. **(B)** HFF cell monolayers were treated with concentration-gradient AT-533 and 17-AAG for 48 h and were manipulated to CPE reduction assay. **(C)** HFF cells were infected with GFP-HSV-1 (MOI = 20) and treated with AT-533 and 17-AAG. After infection for 72 h, the relative fluorescence of GFP-HSV-1 was assessed. Data are shown as means ± SEM. **(D)** HFF cells were infected with HSV-1 (MOI = 20) and treated with AT-533 and 17-AAG at 5 hpi, and the morphology of cells was observed in scale 200 μm at 12 hpi.

CCK-8 assay was performed to examine the cytotoxic effect of two potent Hsp90 inhibitors AT-533 and 17-AAG, on HFF cells. Different concentrations of Hsp90 inhibitors were used. AT-533 exhibited more cytotoxicity than 17-AAG (Figure 1A). TC_50_ (50% toxicity concentrations) of AT-533 and 17-AAG was achieved and listed in Table 1. Hence, to examine the antiviral activities of these Hsp90 inhibitors, CPE (virus-induced cytopathic effect) reduction assay was used (Bag et al., 2012; Mukherjee et al., 2013; Bonvicini et al., 2018). AT-533 (0.125 μM) and 17-AAG (0.20 μM) exhibited reasonable inhibition of induced CPE (Figure 1B). To further clarify the antiviral effect of Hsp90 inhibitors, obtaining IC_50_ (50% inhibition concentrations) in Table 1, an antiviral assay of GFP-HSV-1 was used (Figure 1C; Alvarez et al., 2020; Iqbal et al., 2020). Morphologically, gradient Hsp90 inhibitors exhibited well recovery of HSV-1 cytopathic effects with concentration-dependent (Figure 1D). Overall, Hsp90 inhibitors AT-533 and 17-AAG exhibited significant anti-HSV-1 activity, although AT-533 exhibited more cytotoxicity than 17-AAG.

**TABLE 1:** Cytotoxicity, anti-HSV-1 activity, and TI of AT-533 and 17-AAG.

Hsp90 inhibitors	TC_50_ (μM)	IC_50_ (μM)	TI
17-AAG	14.24 ± 0.26	0.30 ± 0.20	47.90 ± 1.87
AT-533	1.37 ± 0.32	0.05 ± 0.01	25.35 ± 1.03

### Hsp90 Inhibitor Induced the Dissociation Between Hsp90 and UL42

**FIGURE 2:**
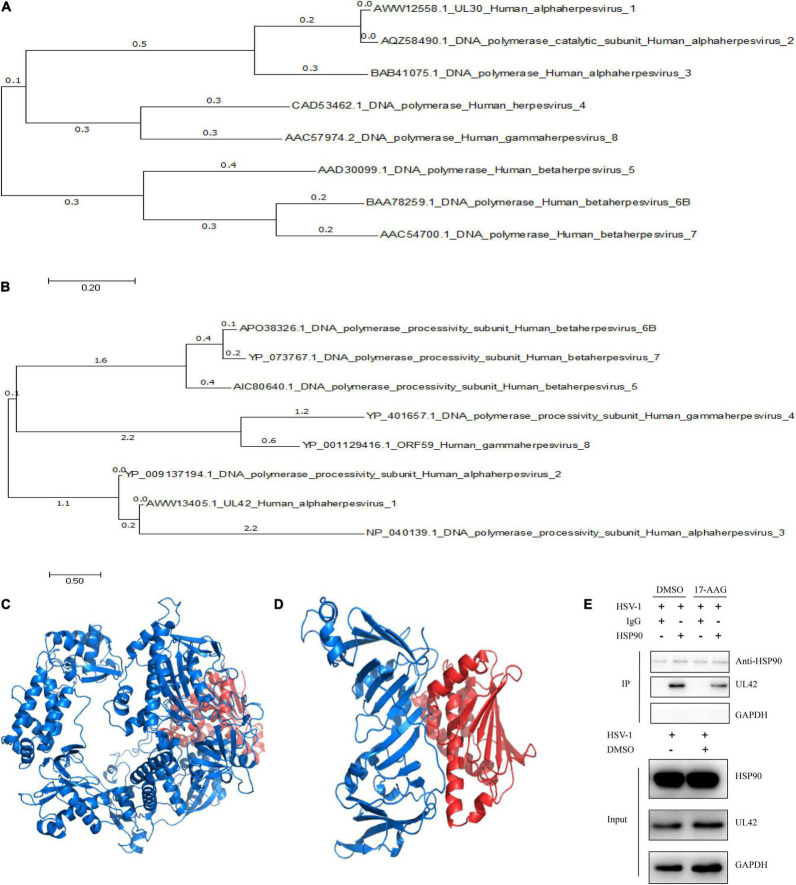
Hsp90 inhibitor-induced the dissociation between Hsp90 and UL42. **(A)** The phylogenetic tree analysis between HSV-1 UL30 and other Herpesviridae virus DNA polymerase catalytic subunits. **(B)** The phylogenetic tree analysis between HSV-1 UL42 and other Herpesviridae viral DNA polymerase processivity factors. **(C)** The cartoon schematic from protein docking stimulation between UL30 and Hsp90. **(D)** The cartoon schematic from protein docking stimulation between UL42 and Hsp90. **(E)** HFF cells were infected with HSV-1 (MOI = 20) and treated with 6.25 μM 17-AAG at 4 hpi. Moreover, the co-immunoprecipitation was manipulated at 12 hpi.

In previous researches, Hsp90 inhibitor geldanamycin induced the abnormal location of HSV-1 DNA polymerase catalytic submit UL30. However, little direct experimental evidence confirmed the interaction between Hsp90 and UL30 or other replication-related proteins (Burch and Weller, 2005). Thus, we further investigated the interaction and function between Hsp90 and HSV-1 replication-related protein. To prepare for the protein modeling and construct the viral proteins model, phylogenetic analysis of HSV-1 DNA polymerase UL30 and UL42 were individually retrieved from GenBank and shown (Figures 2A,B). The crystal structure of DNA polymerase catalytic subunit UL30 (PDB:2GV9) and processivity factor UL42 model from Swissmodel (PDB:1DML) interacted with Hsp90 (PDB:4BQG) were protein docking stimulated (Figures 2C,D). Hence, homology analysis of HSV-1 UL30/42 and other Herpesviridae DNA polymerase was exhibited (Supplementary Tables 2, 3). Altogether, the binding affinity (ΔG), dissociation constant (Kd) (Table 2), and higher number of protein-protein contacts (Supplementary Table 4) between Hsp90 and viral proteins were shown. In conclusion, protein docking stimulation was proved that Hsp90 interacts with both UL30 and UL42. To further confirm the interaction between Hsp90 and HSV-1 DNA polymerase, co-IP assays were performed (Figure 2E). HFF infected with HSV-1 and treated with DMSO were indicated Hsp90 interacts with UL42, and at the same time treated with 6.25 μM 17-AAG were indicated the reducing interaction between Hsp90 and UL42. In short, Hsp90 interacted with HSV-1 DNA polymerase UL42 via protein docking stimulation and co-IP assay. Because of the insufficiency of commercial HSV-1 UL30 antibodies, the interaction between Hsp90 and UL30 still needs further studies.

**TABLE 2:** Binding affinity (ΔG) and dissociation constant (K_d_) predicted values for the interaction between viral proteins and Hsp90β.

Complex	Subtype	ΔG (Kcal mol^–1^)	Mean ΔG (Kcal mol^–1^)	K_d_ (M) at 25°C	Mean K_d_ (M) at 25°C
Hsp90 and BALF5	Hsp90β	–9.3	–9.3	1.4E-07	1.4E-07
Hsp90 and AKT	Hsp90α	–20.0	–15.55	2.3E-15	4.5E-12
	Hsp90β	–11.1		6.7E-09	
Hsp90 and UL30	Hsp90α	–11.6	–13.35	3.2E-09	5.8E-11
	Hsp90β	–15.1		8.3E-12	
Hsp90 and UL42	Hsp90α	–14.2	–13.25	3.9E-11	2.5E-10
	Hsp90β	–12.3		1.0E-09	

### Hsp90 Is Required for the Maintenance of UL42 Stability

**FIGURE 3:**
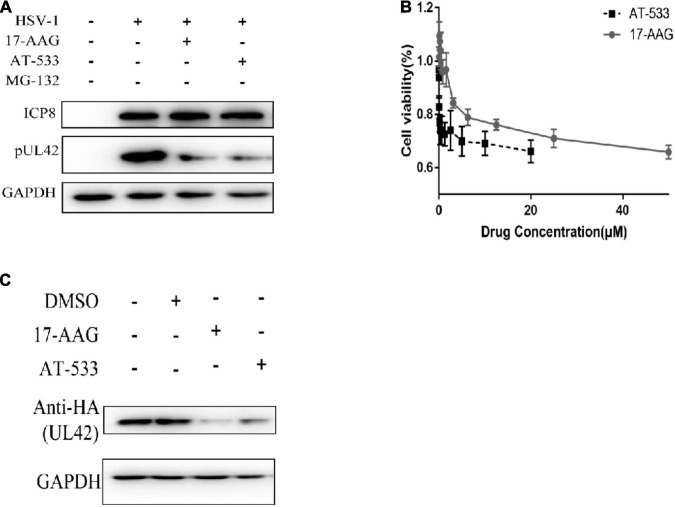
Hsp90 is required for the maintenance of UL42 stability. **(A)** HFF cells were infected with HSV-1 (MOI = 20) and treated with 1.0 μM AT-533 and 6.25 μM 17-AAG at 4 hpi. Western blot was assayed to detect the replication-related viral proteins at 12 hpi. **(B)** HEK293 cell monolayers were treated with concentration-gradient AT-533 and 17-AAG for 48 h and were manipulated to CCK-8 assay. Data are shown as means ± SEM. **(C)** After being transfected with HA-Vector and HA-UL42 plasmid for 48 h, HEK293 cells were refreshed with 2% FBS DMEM containing 1.0 μM AT-533 or 6.25 μM 17-AAG at 6 hpi, and cell lysates were subjected to Western blot assay at 12 hpi.

**FIGURE 4:**
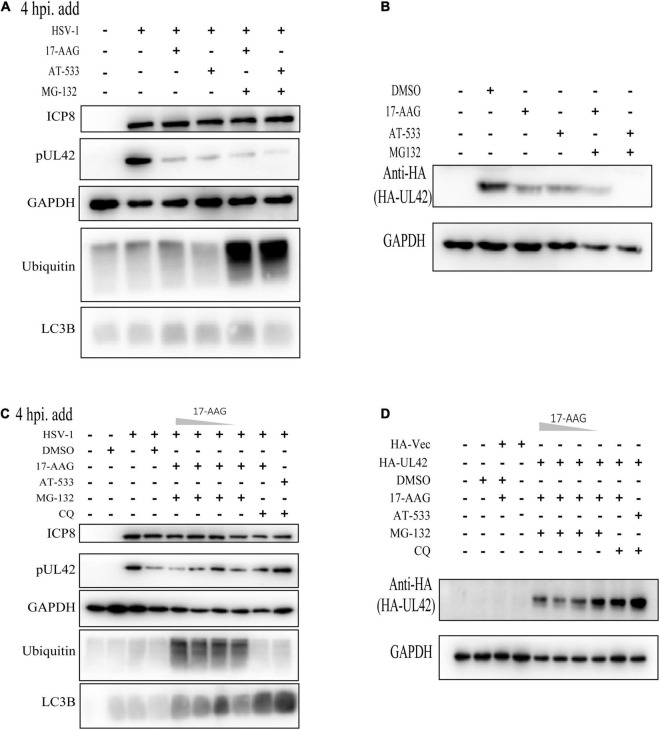
Hsp90 inhibitors mediated autophagy-dependent degradation of UL42. **(A)** HFF cells were infected with HSV-1 (MOI = 20) and treated with Hsp90 inhibitors and proteasome inhibitor MG-132 (10 μM) at 4 hpi, and cell lysates were subjected to Western blot assay at 12 hpi. **(B)** After being transfected with HA-Vector and HA-UL42 plasmid for 48 h, HEK293 cells were refreshed with 2% FBS DMEM containing 1.0 μM AT-533 or 6.25 μM 17-AAG and MG132 (10μM) at 6 hpi, and cell lysates were subjected to Western blot assay at 12 hpi. **(C)** HFF cells were infected with HSV-1 (MOI = 20) and treated with different concentrations of Hsp90 inhibitors and proteasome inhibitor MG-132 (10 μM), autophagy inhibitor CQ (50 μM), and cell lysates were subjected to Western blot. **(D)** After transfected with HA-Vector and HA-UL42 plasmid for 48 h, HEK293 cells were refreshed with 2% FBS DMEM containing 1.0 μM AT-533 or 6.25 μM 17-AAG and MG132 (10 μM), autophagy inhibitor CQ (50 μM) at 6 hpi, and cell lysates were subjected to Western blot assay at 12 hpi.

Western blot was performed to determine whether AT-533 and 17-AAG affect viral DNA replication by affecting UL42 protein stability. To further confirm the key time point of Hsp90 affecting DNA replication, HFF cells are synchronously treated with HSV-1 (MOI = 20) and Hsp90 inhibitors at 4 hpi, as shown in Figure 3A. AT-533 and 17-AAG did not affect the protein stability of ICP8 and affected UL42 indeed. The cytotoxicity of AT-533 and 17-AAG in HEK293T were assayed to prepare plasmids transfection (Figure 3B). AT-533 exhibited higher cytotoxicity than 17-AAG, as shown in Figure 4B. HEK293T cells were transfected with HA-UL42 and HA-Vector plasmid, and at 6 hpi flash, 2% DMEM was added with and without AT-533 or 17-AAG. AT-533 and 17-AAG treatment reduced the HA-UL42 expression and did not affect the transfection of pCMV-HA (Figure 3C). Altogether, Hsp90 inhibitors break the maintenance of viral and over-expressed UL42 stability.

### Hsp90 Inhibitors Mediated Autophagy-Dependent Degradation of UL42

Next, we attempted to identify the specific mechanisms responsible for the Hsp90 inhibition-induced UL42 degradation. In our previous study and those of others, Hsp90 client proteins were degraded via two main ways such as ubiquitination-proteasome pathway and autophagy-induced pathway. Hence, we first assessed the levels of ubiquitinated proteins in HFF cells treated with different Hsp90 inhibitors and found that all treatments did not accumulate the polyubiquitin proteins (Figure 4A). Besides, the proteasome inhibitor MG-132 was treated and did not restore the UL42 reduction of Hsp90 inhibitors inducing. Consistently, HEK293T cells also were treated with or without Hsp90 inhibitors and MG-132, which did not restore the reduction of HA-UL42 (Figure 4B). HFF cells were infected with HSV-1 (MOI = 20) and treated with or without concentration gradient Hsp90 inhibitors and Chloroquine (CQ), an inhibitor of the fusion of the lysosome and autophagosome, to examine whether Hsp90 inhibition-induced UL42 degradation was dependent on the autophagy pathway. CQ could reverse the degradation of UL42 mediated by Hsp90 inhibition (Figure 4C). Similarly, HEK293T cells were transfected with HA-UL42 and treated with Hsp90 inhibitors and CQ. As a result, CQ treatment restored the degradation of UL42 (Figure 4D). Altogether, Hsp90 inhibitors induced autophagy-dependent degradation of viral and over-expressed UL42.

### Hsp90 Inhibitors Restrained ACV-Resistant Strains (HSV-1/153 and Blue) Proliferation

**FIGURE 5:**
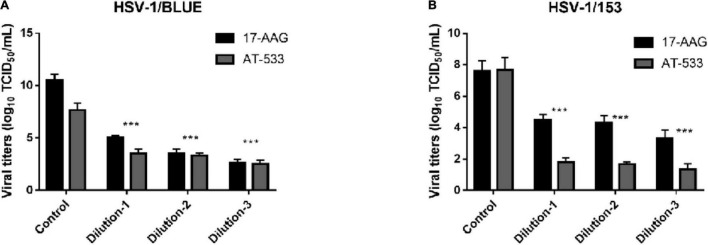
Hsp90 inhibitors restrained ACV-resistant strains (HSV-1/153 and Blue) proliferation. **(A)** HFF cells were infected with HSV-1/Blue (MOI = 1) and treated with Hsp90 inhibitors at 4 hpi, and at 12 hpi, cell lysates were subjected to Vero cells 96 wells-plate to assess viral titer. **(B)** HFF cells were infected with HSV-1/153 (MOI = 1) and treated with Hsp90 inhibitors at 4 hpi, and at 12 hpi, cell lysates were subjected to Vero cells 96 wells-plate to assess viral titer. Dilution 1:17-AAG 3.13 μM, AT-533 2.5 μM, dilution 2:17-AAG 6.25 μM, AT-533 5.0 μM, dilution 3:17-AAG 12.5 μM, AT-533 10.0 μM. Data are shown as means ± SEM. The statistical comparisons above are two-tailed, unpaired Student’s *t*-test with asterisks indicating significance (***P < 0.005).

Finally, we evaluated the potent antiviral activity of AT-533 and 17-AAG on ACV-resistant HSV-1 via performing viral titers determination. Vero cells were infected with HSV-1/153 (MOI = 1) and Hsp90 inhibitors and were extracted to analyze viral titer after 24 hpi (Figure 5A). Indeed, AT-533 and 17-AAG treatment reduced the viral titer of HSV-1/153 with favorable concentration dependence. Therefore, the extraction from above was manipulated for viral DNA detection (Figure 5B). In short, Hsp90 inhibitors restrained ACV-resistant strains (HSV-1/153 and Blue) proliferation.

## Discussion

Several studies have demonstrated that Hsp90 participates in many HSV-1 infectious stages, including early and late stages, which indicates that Hsp90 is a promising candidate for novel drug targets (Wang et al., 2017). Many studies have described the function of Hsp90 in the HSV-1 DNA replication stage. For example, Hsp90 inhibitor geldanamycin reduced viral titers and induced the abnormal location of HSV-1 DNA polymerase catalytic subunit pUL30 (Burch and Weller, 2005; Li et al., 2008). Moreover, Hsp90 interacted with EBV DNA polymerase processivity factor BMRF1 in the cytoplasm to assist complex formation with polymerase catalytic subunit BALF5, indicating the interaction between Hsp90 and its client protein BMRF1 is vital for EBV genome synthesis and disease development (Kawashima et al., 2013). The above studies indicated that Hsp90 plays an indispensable role in the HSV-1 DNA replication stage. However, the function of Hsp90 inhibitors to the interaction of Hsp90 between HSV-1 other replication-related proteins is still unknown.

**FIGURE 6:**
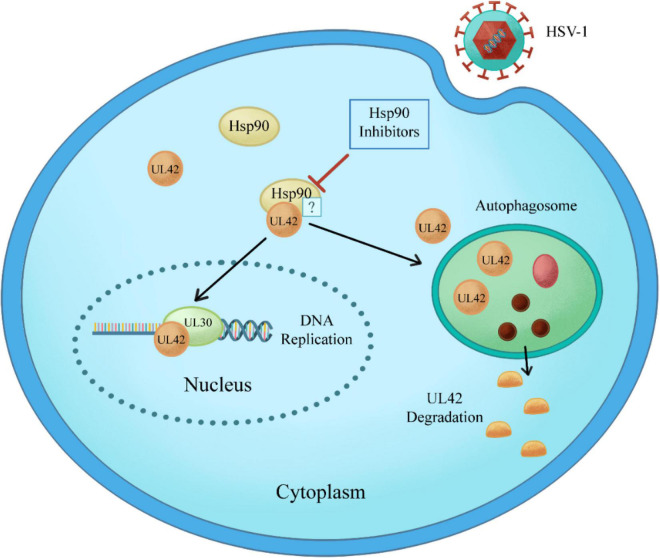
Schematic model of Hsp90 inhibitors regulation of the interaction between Hsp90 and HSV-1 UL42. Specifically, Hsp90 inhibitors disrupt the interaction between Hsp90 and UL42, inducing the degradation in an autophagy-dependent manner.

In this study, CPE assay and GFP-HSV-1 antiviral experiment showed that Hsp90 inhibitors AT-533 and 17-AAG had a significant antiviral effect in a concentration-dependent manner. Recently, three-dimensional and atomic structures of protein complexes can provide useful information for protein engineering, systems biology, drug design, and understanding pathogenic mechanisms (Moal et al., 2018). Therefore, protein docking stimulation was first manipulated in this study, which indicated Hsp90 might both interact with DNA polymerase catalytic subunit UL30 and processivity factor UL42. Hence, the co-immunoprecipitation assay confirmed that UL42 interacted with Hsp90 as its client proteins. Moreover, Hsp90 inhibitors induce the degradation of viral and over-expressed UL42 via autophagy pathway through Western blot assay. As shown in Figure 6, Hsp90 inhibitors induce UL42 dissociation from the Hsp90-UL42 complex and its degradation via autophagosome, contributing to inhibition of HSV-1 proliferation.

The abuse of acyclovir and its derivatives caused the genetic mutation of HSV-1 TK and DNA polymerase UL30. Consequently, ACV-resistant strains bloomed dramatically (Burrel et al., 2010; Andrei and Snoeck, 2013). Notably, Hsp90 inhibitors AT-533 and 17-AAG reduced the viral titer of ACV-resistant strains (HSV-1/153 and HSV-1/Blue). Hence, the HSV-1 DNA polymerase processivity factor UL42 may play an indispensable role in antiviral effects upon ACV-resistant strains, which needs further investigation.

## Data Availability Statement

The datasets presented in this study can be found in online repositories. The names of the repository/repositories and accession number(s) can be found in the article/Supplementary Material.

## Author Contributions

YW, ZR, and SQ formulated the idea of the article and supervised the research. SQ and XH performed the research, analyzed the data, and wrote the manuscript. KL and SQ contributed to the molecular docking. SL, JX, ZW, JJ, and XS revised the data. All authors reviewed the manuscript and approved the final version of the manuscript.

## Conflict of Interest

The authors declare that the research was conducted in the absence of any commercial or financial relationships that could be construed as a potential conflict of interest.

## Publisher’s Note

All claims expressed in this article are solely those of the authors and do not necessarily represent those of their affiliated organizations, or those of the publisher, the editors and the reviewers. Any product that may be evaluated in this article, or claim that may be made by its manufacturer, is not guaranteed or endorsed by the publisher.

## Footnotes

1
http://zdock.umassmed.edu/

2
http://www.lephar.com/index.htm

3
http://www.rcsb.org/

4
http://zinc.docking.org/
