# Supplementary material for: Hsp90 Inhibitors Prevent HSV-1 Replication by Directly Targeting UL42-Hsp90 Complex
Source: Front Microbiol. 2022 Feb 3;12:797279. doi: 10.3389/fmicb.2021.797279 (PMC8851068; doi:10.3389/fmicb.2021.797279)
Supplement: Supplementary file 1 [file Table_1.DOC]

Supplementary Material

**Supplementary Tables**

**Table S1.** Summarization of PCR Amplification Primer used in this research

| **Name of plasmids** | **Primer sequences** |
| --- | --- |
| p3xFLAG-UL30 | Forward：ggatgacgatgacaagctt  ATGTTTTCCGGTGGCGGC |
|  | Reverse:tcatgcatgcctgtggatcc  TTTATTGTAAAATGAGGGACATCAGC |
| pCMV-HA-UL42 | Forward：gccatggaggcccgaattc  ATGACGGATTCCCCTGGCG |
|  | Reverse:tctggatccccagcggccgc  TTTATTTACATTAACCCGGGATGG |

**Table S2.** Homology analysis of HSV-1 U_L_30 and other Herpesviridae DNA polymerase catalytic subunit

| **Description** | **Query Cover** | **Percent Identity** |
| --- | --- | --- |
| HSV-2 U_L_30 | 100% | 90.40% |
| HHV-3/VZV ORF28 | 95% | 55.42% |
| HHV-4/ZBV BALF5 | 92% | 41.75% |
| HHV-5/HCMV U_L_54 | 82% | 34.81% |
| HHV-6 Pol | 92% | 31.21% |
| HHV-7 Pol | 93% | 31.81% |
| HHV-8/KSHV Pol | 95% | 55.42% |

**Table S3.** Homology analysis of HSV-1 U_L_42 and other Herpesviridae DNA polymerase processivity factor

| **Description** | **Query Cover** | **Percent Identity** |
| --- | --- | --- |
| HSV-2 U_L_42 | 100% | 71.14% |
| HHV-3/VZV ORF16 | 56% | 25.44% |
| HHV-5/HCMV UL44 | 9% | 40% |
| HHV-6 p41 | 2% | 50% |
| HHV-7 U27 | 10% | 32.50% |
| HHV-8/KSHV PF-8 | 19% | 39.58% |

**Table S4.** Number of protein-protein contacts (PPC) between viral proteins and Hsp90β

| **Complex** | Hsp90 & BALF5 | Hsp90 & AKT | | Hsp90 & U_L_30 | | Hsp90 & U_L_42 | |
| --- | --- | --- | --- | --- | --- | --- | --- |
| **Subtype** | Hsp90β | Hsp90α | Hsp90β | Hsp90α | Hsp90β | Hsp90α | Hsp90β |
| ICs charged-charged | 2 | 14 | 12 | 12 | 11 | 13 | 6 |
| ICs charged-polar | 8 | 25 | 15 | 23 | 19 | 15 | 8 |
| ICs charged-apolar | 8 | 81 | 20 | 42 | 45 | 37 | 25 |
| ICs polar-polar | 6 | 4 | 5 | 4 | 6 | 6 | 1 |
| ICs polar-apolar | 25 | 25 | 21 | 12 | 33 | 24 | 20 |
| ICs apolar-apolar | 28 | 32 | 15 | 21 | 29 | 33 | 23 |
| Total Number of Contacts | 77 | 181 | 88 | 114 | 143 | 128 | 83 |
| Mean Number of Contacts | 77 | 134 | | 128 | | 105 | |
